# Low Concentration of Silver Nanoparticles Not Only Enhances the Activity of Horseradish Peroxidase but Alter the Structure Also

**DOI:** 10.1371/journal.pone.0041422

**Published:** 2012-07-27

**Authors:** Zoheb Karim, Rohana Adnan, Mohd Saquib Ansari

**Affiliations:** 1 School of Chemical Sciences, Universiti Sains Malaysia, Penang, Malaysia; 2 School of Biomedical Sciences, Shaheed Rajguru College of Applied Sciences for Woman Delhi University, Delhi, India; University of Helsinki, Finland

## Abstract

Chemical synthesis of Ag-NPs was carried out using reduction method. The reduction mechanistic approach of silver ions was found to be a basic clue for the formation of the Ag-NPs. The nanoparticles were characterized by UV-vis, FT-IR and TEM analysis. We had designed some experiments in support of our hypothesis, “low concentrations of novel nanoparticles (silver and gold) increases the activity of plant peroxidases and alter their structure also”, we had used Ag-NPs and HRP as models. The immobilization/interaction experiment had demonstrated the specific concentration range of the Ag-NPs and within this range, an increase in HRP activity was reported. At 0.08 mM concentration of Ag-NPs, 50% increase in the activity yield was found. The U.V-vis spectra had demonstrated the increase in the absorbance of HRP within the reported concentration range (0.06–0.12 mM). Above and below this concentration range there was a decrease in the activity of HRP. The results that we had found from the fluorescence spectra were also in favor of our hypothesis. There was a maximum increase in ellipticity and α-helix contents in the presence of 0.08 mM concentration of Ag-NPs, demonstrated by circular dichroism (CD) spectra. Finally, incubation of a plant peroxidase, HRP with Ag-NPs, within the reported concentration range not only enhances the activity but also alter the structure.

## Introduction

Nanotechnology, an interdisciplinary research field involving chemistry, engineering, biology, and medicine, has great potential for early detection, accurate diagnosis and personalized treatment of cancer and other diseases [1]. Nanoparticles (NPs) are typically smaller than several hundred nanometers in size, comparable to large biological molecules such as enzymes, receptors and antibodies. With the size of about one hundred to ten thousand times smaller than human cells, these nanoparticles can offer unprecedented interactions with biomolecules both on the surface and inside the cells and biological molecules. The most well studied nanoparticles are quantum dots [2], [3], carbon nanotubes [4], paramagnetic nanoparticles [5], liposomes [6], gold nanoparticles [7] and many others [8]. It seems that these NPs might take center stage in many key future technologies because of recent efforts in fabricating these nanosized structures into predefined superstructures. In order to meet the wide scope of nanomaterials, an overwhelming number of protocols have been exploited for their synthesis, but unfortunately, most of them are capital intensive, inefficient in material and energy use and often pose health hazards as toxic chemicals. Therefore, there is a need to develop safe, reliable, nontoxic, cost effective, clean and ecofriendly methods for the preparation of NPs.

The interaction of enzymes with ligands offered stimulating opportunities for a wide variety of applications in the field of biotechnology and medicine [9], [10]. Generally, proteins undergo structural changes when interacting with ligands. Novel NPs (silver and gold) serve as novel entities for the binding of protein due to their large surface to volume ratio, high biocompatibility, non-toxicity, chemical stability and ease of preparation [11], [12]. Immobilization of proteins onto solid surfaces strongly depends on the nature of the protein and surface geometry and physicochemical characteristics of the solid surface. Binding of enzymes directly on the naked surface of bulk materials may result in their denaturation and loss of activity. However, enzymes immobilization onto NPs may alter their catalytic activity [13]. Various specific and nonspecific interactions such as electrostatic, hydrogen bonding and hydrophobic interactions are involved in the immobilization of protein on NPs which affect the structure and stability of proteins [14].

The present study focused on the preparation of the silver nanoparticles (Ag-NPs) by sodium borohydride reduction of silver nitrate with a citrate stabilizer. The method ensured that the NPs provided an intense surface plasmon band that could be used in the colorimetric assay. The negative citrate ions surrounding the Ag-NPs provide enough electrostatic repulsion to overcome the attractive hydrophobic and van der Waals forces and, in doing so, caused the Ag-NPs to remain stable and dispersive in an aqueous solution. The characterization of Ag-NPs was performed by UV-vis, FT-IR and TEM analysis. The low concentration of the Ag-NPs enhances the catalytic activity of the horseradish peroxidase (HRP) enzyme. Structural changes in enzymes after binding with Ag-NPs were analyzed by UV–vis, fluorescence, circular dichroism (CD) spectroscopy and FT-IR spectral analysis.

## Materials and Methods

### Materials

Horseradish peroxidase, trisodium citrate, sodium borohydride and silver nitrate *(*AgNO_3_ *)* were purchased from Sigma-Aldrich Co. (USA). All other chemicals and reagents were of analytical grade and used without further purification. All the experiments were performed in 100 mM sodium acetate buffer, pH 5.5 except where specified. The concentration of protein was determined spectrophotometrically using E1% 280 nm = 2.493 on a Perkin Elmer, Lambda 25 spectrophotometer and alternatively by a protein dye binding method [15].

### Synthesis of silver nanoparticles

Synthesis of Ag-NPs was done as described by Chen et al. [16] with some small modifications. AgNO_3_ aqueous solution (1 mL, 0.01 M) and trisodium citrate dehydrate aqueous solution (1 mL, 0.03 M) were added to Ultrapure water (97 mL) and sodium borohydride aqueous solution (1 mL, 1.79 mg/mL) was dropped into the solution with stirring. The formation of the Ag-NPs was confirmed when the solution changed to a yellow color. After about 20 min, precipitate was collected by centrifugation, washed with ethanol/water (90:10 v/v) and freeze dried. The resulting powder was resuspended in distilled water to achieve a concentration of 10.0 mM for further analysis.

### Immobilization of HRP with Ag-NPs

The obtained Ag-NPs were dissolved in sodium acetate buffer, pH 5.5 at 4°C with defined unit of HRP. Concentrations of Ag-NPs were varied by keeping the unit of HRP constant to achieve the best Ag-NPs-HRP ratio and hence determine the minimum concentration of Ag-NPs which enhanced the activity of HRP. The mixture was homogenized at 1500 rpm for 1 h at 4°C to achieve the best loading capacity of HRP on the Ag-NPs. The resulting solution was then centrifuged at 8000 rpm for 15 min to separate the unbound Ag-NPs. The collected pellet was freeze-dried for 24 h. This Ag-NPs-HRP complex preparation was stored at 4°C for further used.

The Ag-NPs-HRP complex was crosslinked by 0.5% glutaraldehyde at pH 5.5 for 1 h at 4°C. Glutaraldehyde treated complex was incubated with 0.01% (v/v) ethanolamine for 90 min at 4°C in order to stop crosslinking. Excess ethanolamine was removed by centrifugation and crosslinked Ag-NPs-HRP complex was washed thrice [17], [18]. Finally crosslinked Ag-NPs-HRP complex was suspended in 100 mM sodium acetate buffer, pH 5.5 and stored at 4°C for further used.

### Instrumentations of native and immobilized Ag-NPs

A stock solution of HRP (5.0 mM) was prepared in 100 mM sodium acetate buffer, pH 5.6 and diluted with the same buffer as necessary. The HRP concentration was kept constant at 20.0 µM for UV-vis and FT-IR analysis; but at 5.0 µM and 3.0 µM for CD and fluorescence measurements, respectively, while varying the concentrations of Ag-NPs as mentioned in the Table 1. The reaction mixture was equilibrated for the optimal incubation time at 25°C for 1 h. Subsequently, UV-vis absorption, FT-IR, fluorescence and CD spectra of the solution were recorded to monitor the interaction between HRP and Ag-NPs.

**Table 1:** Effect of Ag-NPs concentrations on the activity of HRP.

Cons. of Ag-NPs	Enzyme Loaded, X (U)	Enzyme activity in wash Y (U)	Activity bound on Ag-NPs (U)			Percent activity yield, B/A 100
			Theoretical (X-Y = A) (A)	Actual (B)	Effectiveness factor (B/A) (η)	
0.04 mM	3000	198	2802	2732	0.97	97
0.06 mM	3000	238	2762	3672	1.32	132
0.08 mM	3000	245	2755	4135	1.15	150
0.10 mM	3000	300	2700	3050	1.12	112
0.12 mM	3000	319	2681	2747	1.02	102
0.14 mM	3000	340	2660	2554	0.96	96
0.16 mM	3000	356	2648	2447	0.92	92

The immobilization of HRP with different concentrations of Ag-NPs was performed as described in the text. The concentration range that was selected for the immobilization was 0.04–0.16 mM of silver nanoparticles.

### UV-visible spectra

UV-visible spectra were recorded by Perkin Elmer, Lambda 25 spectrophotometer. The measurements were done in a 10 mm quartz cell. Enzyme concentration was kept constant at 20.0 µM in each reaction mixture while the NPs concentration varied from 0.04 to 0.16 mM. Unless otherwise stated, the UV–vis spectra were recorded in a wavelength range of 210–410 nm. For sample measurements, the baseline was always set with a relevant blank. The final spectrum is an average of three independent measurements.

### FT-IR spectra

The Fourier transform infrared (FT-IR) spectral studies were performed using KBr pelleting technique with Perkin Elmer System 2000 instrument in range of 400–4000 cm^−1^. FT-IR analysis was carried out to determine the variation of the functional groups present in the native compounds and in the prepared complex.

### TEM analysis

Freshly prepared suspensions of all samples in 1-propanol were air-dried and sputter-layered with gold. TEM of the prepared particles was carried out using a Leica Cambridge S360 scanning microscope (USA).

### Fluorescence spectra measurement

Fluorescence analyses were performed on a Shimadzu Spectrofluorophotometer (RF-5301) equipped with a DR-3 data recorder. The fluorescence spectra were measured at 25±0.10°C with a 1.0 cm path length. Both excitation and emission slits were set at 5.0 nm. The intrinsic fluorescence of a protein sample was measured by exciting at 280 nm and emission spectra were recorded in the range of 300–500 nm.

### CD spectroscopy

The CD measurements were carried out on Jasco Spectropolarimeter (J-720). The instrument was calibrated with *d*-10-camphorsulfonic acid. All the CD measurements were carried out at 25°C using a thermostatically controlled cell holder attached to a Neslab RTE-110 water bath with an accuracy of 0.10°C. Far-UV CD spectra were measured at a protein concentration of 7.0 µM. The path length of the cell was 1.0 mm. Mean residue ellipticity (MRE) was calculated as given below.


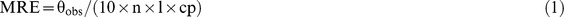


where θ_obs_ is the CD in milli degrees, n is the number of amino acid residues (483), *l* is the path length of the cell and cp is the mole fraction.

The % *α*-helical content was calculated from the MRE values at 222 nm using the following equation as described by Chen et al. [19].


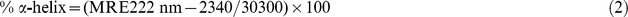


### Assay of peroxidase activity

The activity of peroxidase was estimated from the change in the optical density (*A*_460_ nm) in 100 mM sodium acetate buffer, pH 5.0 at 40°C by measuring the initial rate of oxidation of 6.0 mM *o*-dianisidine HCl by 18 mM H_2_O_2_ [20].

One unit (1.0 U) of peroxidase activity was defined as the amount of enzyme protein that catalyzed the oxidation of 1.0 µ mole of *o*-dianisidine HCl per min at 40°C into colored product (ε_m_ = 30000 M^−1^ cm^−1^).

### Statistical analysis

Each value represents the mean of three independent experiments performed in duplicates, with average deviations, less than 5%. Data expressed in various studies was plotted using Sigma Plot-10.0 and Microsoft Excel 2003. *P*-values less than 0.05 were considered statistically significant, expressed as mean with standard deviation of error (±).

## Results and Discussion

### Biophysical characterization of Ag-NPs

There are various methods available to synthesize nanomaterials. The chemical synthesis of NPs offered several advantages like lower processing temperature, better homogeneity, controlled stoichiometry and flexibility of forming nanoparticles. Ag-NPs have attracted much attention owing to their remarkable physicochemical properties like high surface area, high electrical conductivity, good chemical stability and significant mechanical strength, therefore these NPs have been widely applied for the immobilization of wide range of biomolecules [21].

### UV-vis absorption spectra

**Figure 1:**
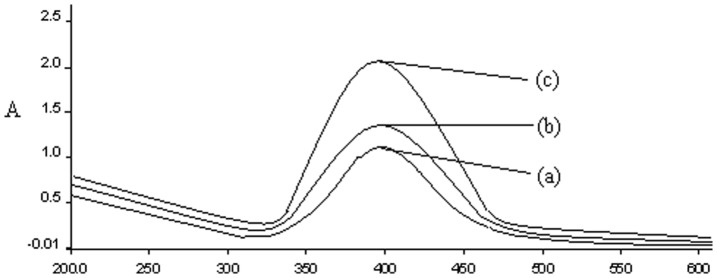
UV-vis spectra of Ag-NPs with time; (a) 20 (b) 40 and (c) 60 min. The synthesis of Ag-NPs was performed as described in the text.

UV-VIS absorption spectra have been proved to be quite sensitive to the formation of silver nanoparticles because silver nanoparticles exhibit an intense absorption peak due to the surface plasmon (it describes the collective excitation of conduction electrons in a metal) excitation. To characterize the synthesized Ag-NPs, the U.V-vis spectra were recorded using double beam spectrophotometer (as mentioned previously). The change in the color from white to yellow illustrates the formation of the silver nanoparticles with the peak at 400 nm and was further confirmed the formation of Ag-NPs by chemical reduction method. Fig. 1 shows the UV-Vis spectra of the Ag-NPs in the range of 200–600 nm. The absorption band in visible light region (340–550 nm, plasmon peak at 400 nm) is typical for Ag-NPs. The plasmon peak and the full-width of half-maximum depend on the extent of aggregation [22]. To monitor the stability of the silver NPs aggregation, we have measured the absorption for different periods of time as shown in the same figure. There was no obvious change in peak position with different time intervals, except for the increase in the absorbance which indicates that amount of Ag-NPs increases with time. The stable position of the absorbance peak indicates that no new aggregates formed.

### TEM spectra

**Figure 2:**
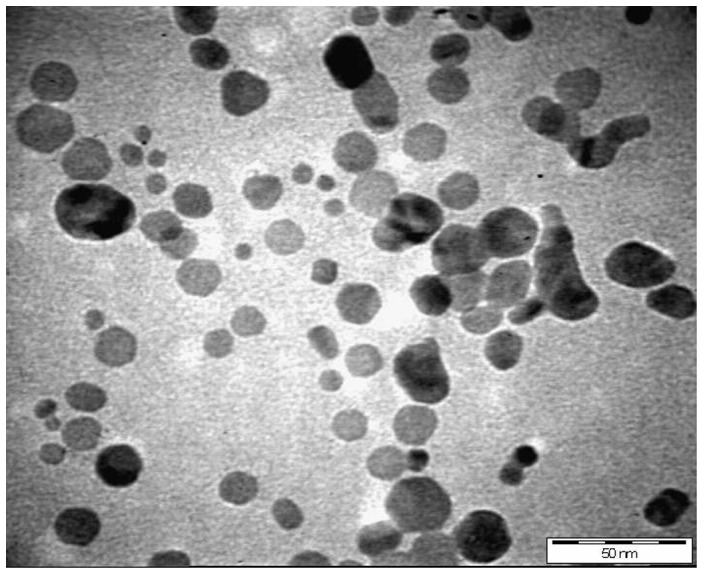
TEM spectra of prepared Ag-NPs. The TEM spectra were monitor as described in the text with magnification of 500000×.

**Figure 3:**
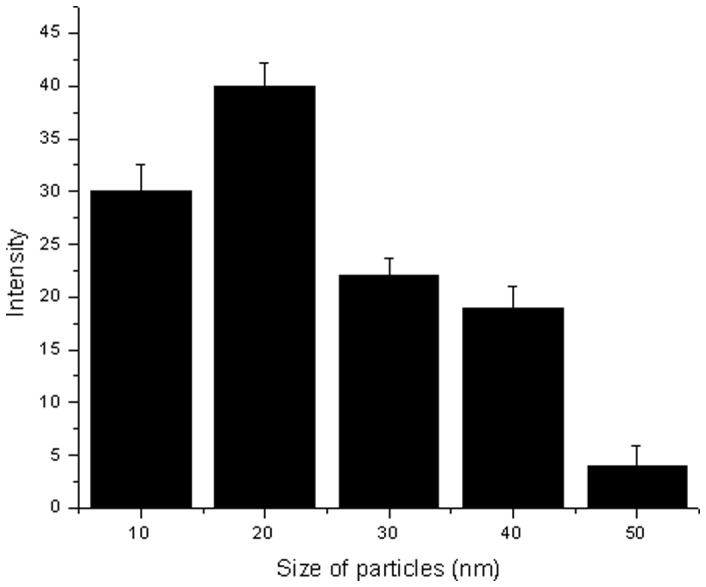
Particle size distributions of Ag-NPs prepared using chemical reduction method. The figure confirms the formation of approx 20 nm silver nanoparticles by chemically reduction method.

TEM images of Ag-NPs given in Fig. 2 revealed that nearly spherical Ag-NPs are distributed uniformly. The images show agglomerates of small grains and some dispersed nanoparticles. Aggregation of the NPs observed was due to the overlapping of some bigger particles with the smaller particles. The particle size histograms of silver particles (Fig. 3) illustrates that the particle size range from 8 to 50 nm with the mean diameter of 20 nm. The results suggest that the chemical reduction process did not change the morphology of the Ag-NPs even though the size increased. Recently, Maribel et al. [23] synthesized silver nanoparticles with reduction method. The TEM micrographs indicate that the Ag-NPs consist of well dispersed agglomerates of grains with a narrow size distribution (40 and 60 nm), whereas the radius of the individual particles were found to be between 10 and 20 nm.

### Interaction studies of Ag-NPs with HRP

Recently, it has been reported that peroxidases can be used in various scientific fields [17]. Several methods have been used for the immobilization of peroxidases from various sources with different physical as well as chemical supports but most of the immobilized enzyme preparations show low activity, stability and effectiveness due to the leaching of enzymes from the support. These factors were affecting the application of the immobilized enzyme system in various fields as such enzymes preparations could not meet the requirements for the delivery of site specific drugs into the human body [7]. However, among the techniques used for the immobilization of enzymes, adsorption has several merits over the other known methods. It is a simple procedure and can be exploited for the direct immobilization of enzymes even from the crude cell homogenates. For the first time in the field of bio-nanotechnology, we have reported the immobilization of HRP with different concentration of chemically formed Ag-NPs by reduction method. To stop the leaching of the enzyme from the support (Ag-NPs), crosslinking with 0.5% glutaraldehyde at pH 5.5 has been also performed. Earlier researchers reported that the crosslinking of enzymes by glutaraldehyde with their support make the immobilized preparation more stable [24].

### FT-IR spectra of native and HRP bound Ag-NPs

FT-IR is a powerful tool for identifying types of chemical bonds in a molecule by producing an infrared absorption spectrum that is like a molecular “fingerprint” [25]. The wavelength of light absorbed is characteristic of the chemical bond as can be seen in this annotated spectrum. Because the strength of the absorption is proportional to the concentration, FT-IR can be used for quantitative analyses. The FT-IR measurement can also be utilized to study the presence of a protein molecule in the solution, as the FT-IR spectra in the 1400–1700 cm^−1^ region provides information about the presence of C = O and N-H groups [26]. The amide linkages between amino acid residues in polypeptides and proteins give rise to well known signatures in the infrared region of the electromagnetic spectrum. The position of the amide I and II bands in the FT-IR spectra of proteins is a sensitive indicator of conformational changes in the protein-secondary structure [27], [28].

**Figure 4:**
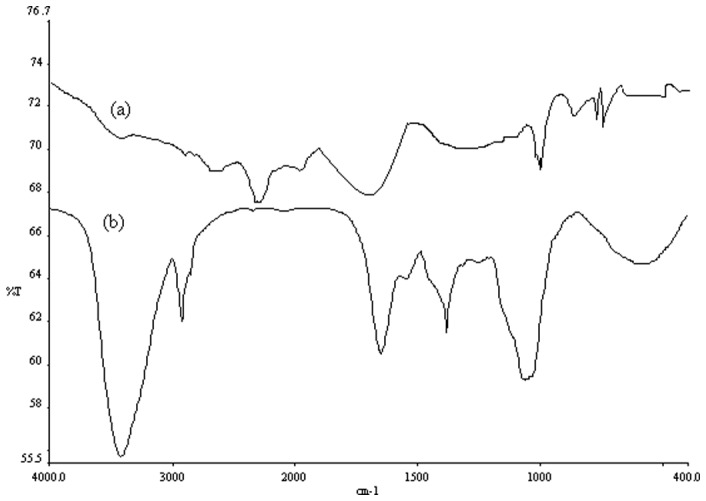
FT-IR spectra of chemically synthesized Ag-NPs and bound Ag-NPs with HRP. FT-IR spectra were measured as described in the text. The freshly prepared native Ag-NPs and bound with HRP samples were recorded using KBr pelleting technique with Perkin Elmer System 2000 instrument in range of 400–4000 cm^−1^. Figures demonstrate the native Ag-NPs (a) and bound Ag-NPs with HRP (b).

The binding of HRP with Ag-NPs was confirmed by FT-IR analysis. The sample exhibited a peak at 554 cm^−1^ which could be assigned to the stretching vibration of the Ag–O framework (Fig. 4a). The band intensity of protein at 1643 cm^−1^ showed the bending vibration of the N–H group which decreased after the adsorption of the enzyme on the NP surface (Fig. 4b). Other characteristic bands at 1110 and 1036 cm^−1^ are due to the stretching and bending vibrations of aliphatic amines. The strong absorption peak at 1212 cm^−1^ (Fig. 4a) was due to interaction of the NH group of HRP with the Ag–O group of NPs [29], [30]. The intense and broad peak at 3506 cm^−1^ and 1638 cm^−1^ (Fig. 4b) can be attributed to the O–H vibration and water absorbed on the sample surface [31]. The bands at 1650 and 1450 cm^−1^ are due to C = O and N-H stretching vibrations present in the amide linkages of the proteins, respectively. The positions of these bands are close to reported literature values for native proteins [26].

### Immobilization of HRP with different concentrations of Ag-NPs

The binding of HRP (3000 U) with increasing concentration of Ag**-**NPs (0.04**–**0.16 mM) has been exploited for immobilization of peroxidase from horseradish at sodium acetate buffer having pH 5.5. The maximum theoretical binding of peroxidase (2802 U) was found at 0.04 mM Ag-NPs (Table 1), followed by 0.06 mM and 0.08 mM Ag**-**NPs, respectively. Table 1 further illustrates the actual binding of enzyme with various concentrations of Ag-NPs; at 0.08 mM concentration of Ag-NPs, the most effective binding activity (4135 U) has occurred having very high effectiveness factor of ‘*η*’ equals to 1.15. Thus, concentrations of Ag-NPs from 0.06–0.12 mM enhance the binding activity of HRP on Ag-NPs, below and above the mentioned concentrations decrease the binding activity of HRP. Now it can be concluded that at the low concentrations of Ag**-**NPs, the activity of the immobilized HRP was enhanced due to the changes in the structure of HRP. These findings have been further supported by CD and fluorescence spectra, described in the later section. Effectiveness factor of an immobilized enzyme is a measure of internal diffusion and reflects the efficiency of the immobilization procedure [24]. In this case, the yield of immobilization was quite superior over other methods used for the immobilization [18], [24]. The prepared immobilized preparation had non-covalent interactions with the support. In a published article, the immobilization of different enzymes with support had been discussed. The main forces that were responsible for the stability of immobilized preparations were ionic, hydrogen, van der Waals and hydrophobic interactions [32].

Ho et al. [33] assayed many pure enzymes in buffer solution and found that, generally all mentioned enzymes has decreased activities, however, lysozyme and pepsin showed increasing activities. Our findings are also in support of recent published articles in which the nanogold/microgel particles were conjugated with enzymes; HRP and urease. It was found that under identical assay conditions, the enzyme/nanogold/microgel systems exhibited enhanced biocatalytic activities over free enzymes in solution, especially at lower enzyme concentrations. Moreover, HRP/nanogold/microgel systems showed higher activities at various pH, temperatures and storage stability as compared to free enzymes [34].

### U.V-vis spectra in support of our hypothesis

**Figure 5:**
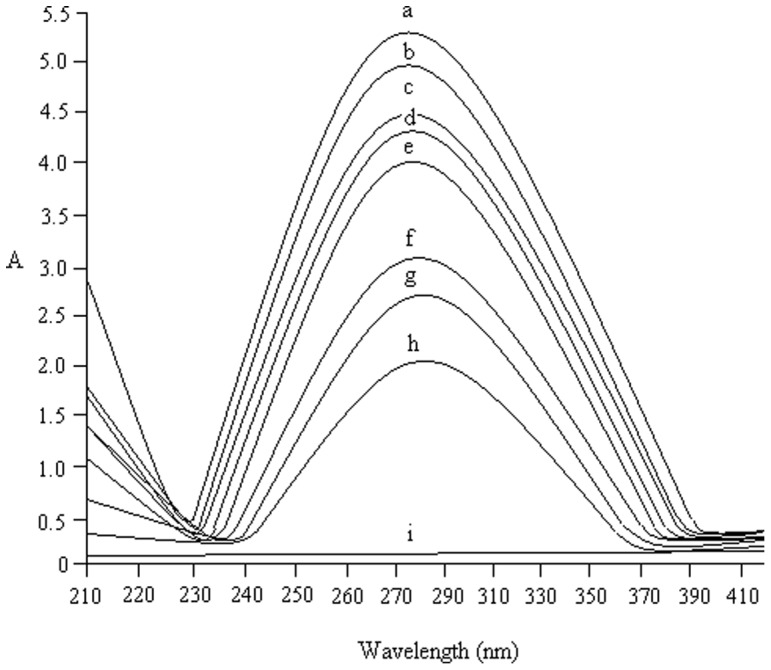
U.V-vis spectra of HRP with different concentration of Ag-NPs. The U.V-vis spectra of the interaction of HRP with different concentration of Ag-NPs were recorded as mentioned in the text. The concentration range was from 0.04 to 0.16 mM of Ag-NPs. The alphabets presents different concentrations of nanoparticles; a- 0.08 mM, b-0.06 mM, c-0.10 mM, d-0.12 mM, e-enzyme, f-0.04 mM, g- 0.14 mM, h-0.16 mM and i-nanoparticle.

UV–vis spectroscopy is an effective and simple tool to explore structural change in protein molecules [35], [36]. The UV–vis spectra obtained by the NP titration experiments demonstrate that the protein microenvironment at pH 5.5 was perturbed (Fig. 5). Upon increasing concentrations of Ag-NPs, a consecutive regular increase in the absorbance maxima of protein at 280 nm is observed. Fig. 5 further illustrates the increase in the activity profile of HRP with low concentrations of silver nanoparticles as demonstrates in Table 1. The maximum increase in the HRP activity occurred in the presence of 0.08 mM concentration of Ag-NPs. This might be due to the partial or full adsorption of enzyme on the surface of the NPs. These results indicate that there is an interaction between Ag-NPs and HRP through ground state complex formation [37].

In this system, the binding process occurred directly upon activation of the particle surface. It can be argued that the direct binding between the protein and NPs exhibited a greater activation to a medium with given pH. This medium appeared even more constraining to the enzyme and placed the enzyme in an electrostatic state that might affect the activity. Non-covalent binding was expected to provide the enzyme with the protection against structural denaturation due to the unfavorable solvent-protein interactions, and thus result in activation effect [32], a possible reason for a better activity of the bound enzyme with low concentration of NPs.

### Fluorescence spectra measurement

**Figure 6:**
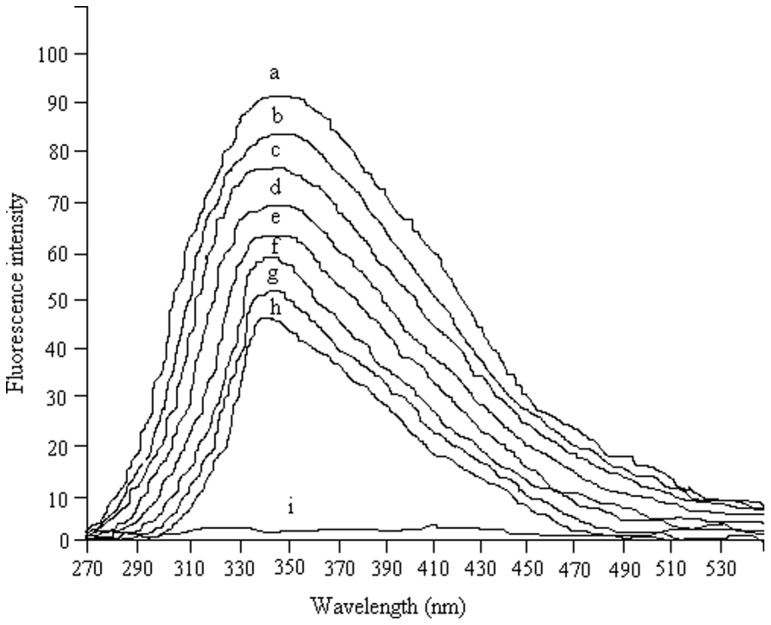
Fluorescence spectra measurement. Fluorescence spectra were recorded as described in the text with the different concentration of Ag-NPs. For alphabetic representation please refer to Fig. 5. The increase in the fluorescence intensity was in the support of our hypothesis.

Fluorescence spectroscopy has been shown to be a very useful technique for studying structure and dynamics in protein. Environmental changes resulting from conformational changes in the tertiary structure of proteins can be measured by intrinsic fluorescence spectroscopy [38]. The intrinsic fluorescence emission from tryptophan residue in proteins was an in-built probe and was sensitive to the microenvironment surrounding the fluorophore residue [39]. Therefore, the change in the tertiary structure of HRP protein could be determined by intrinsic emission spectroscopy and fluorescence intensity. Fig. 6 depicts the fluorescence emission spectra of HRP at the excitation wavelength of 293 nm (determined by present study). All samples were measured immediately after treatment with different concentrations of Ag-NPs at 4°C. The fluorescence emission intensity of untreated HRP was in the middle of concentration range, because the tryptophan fluorescence was quenched due to the intra-molecular tryptophan-heme energy transfer in native HRP [38], [40]. The fluorescence emission spectra of HRP subjected to different concentrations of Ag-NPs treatment showed a great change of the emission maxima and the maximum fluorescence intensity had occurred, indicating a change in the tertiary structure of HRP. The emission maximum wavelength, λ_max_, of untreated HRP was observed at 334 nm, while the λ_max_ for Ag-NPs-treated HRP varies in respond to the concentration of Ag-NPs. A wide span of red-shift as well as blue shift in the emission spectra of the Ag-NPs treated HRP shows the increase as well as decrease of the HRP activity due to the change in the tertiary structure. The red shift occurred in the HRP emission spectra with 0.04, 0.14 and 0.16 mM Ag-NPs but on the other hand there was a blue shift within the range of 0.06–0.12 mM of chemically prepared Ag-NPs, indicating that the tryptophan surroundings changed to a more polar environment upon Ag-NPs treatment. Above and below 0.08 mM concentration of Ag-NPs there was a decrease in the fluorescence intensity (Fig. 6). NPs having an average diameter of 20 nm effectively quench HRP as the mentioned concentrations of silver nanoparticles, as evidenced by the progressive decrease in the emission maximum intensity. This quenching effect indicates that Ag-NPs strongly interact with the chromophore residues of HRP. The tendency of red shift to occur in the protein upon binding of NPs suggests that the interaction was specific and involved only a limited part of protein. The changes in the fluorescence emission maxima suggest the solvent exposure of some of the tryptophan residue that was in solvent hydrophobic core of the protein under native conditions. Quenching of fluorescence emission spectra by metallic NPs has also been earlier investigated [41]. The change of protein intrinsic fluorescence intensity could reflect its local conformational change. After NPs treatment, activity of HRP became smaller but the fluorescence intensity of HRP became stronger. It seems that the change of intrinsic relative fluorescence intensity of HRP activity is consistent with that of different concentrations of NPs. Theorizing the mechanism that causes this is beyond the scope of the present study.

### CD spectra measurement

CD is a valuable spectroscopic technique for studying protein conformation in solution because many common conformational motifs containing α-helices, β-pleated sheets, and poly-L-proline II-like helices and turns, have characteristic far UV(190–245 nm) CD spectra [42], and directly characterize the change of protein secondary conformation [43].

**Figure 7:**
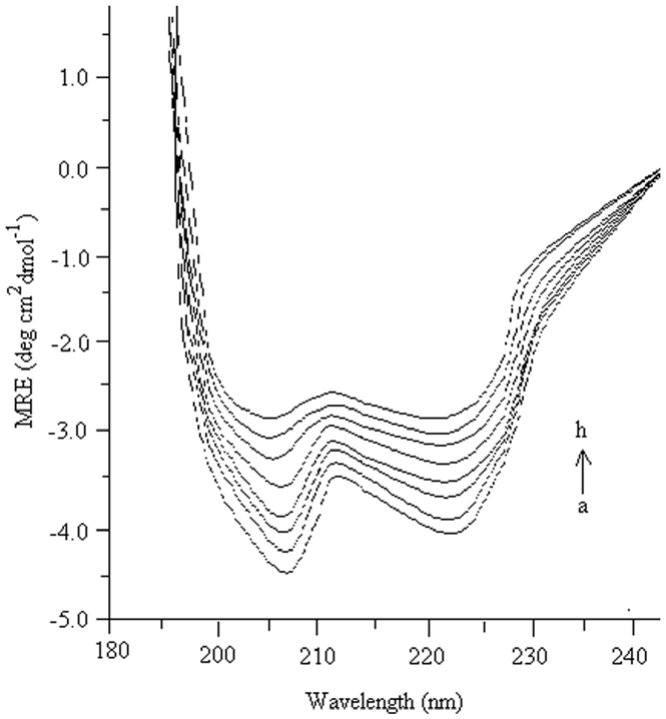
CD spectra of HRP with different concentration of Ag- NPs. The far UV-CD spectra of all the samples were measured immediately after treatment with different concentration of Ag-NPs at 4°C as mentioned in the text. For alphabetic reference refer Fig. 5.

The far UV-CD spectra of all the samples were measured immediately after treatment with different concentrations of Ag-NPs at 4°C. Fig. 7 presents the CD spectra of HRP in the region of peptide [40], bond absorption (190–245 nm) as a function of NPs at 55°C. It is commonly known that two negative peaks, at 208 and 222 nm, are the characteristic of a protein with a large amount of α-helix secondary structure [44], and their intensity reflects the amount of helical structure in protein [45]. As shown in Fig. 7, HRP had double negative peaks in the far UV-CD spectra at around 208 and 222 nm. Veitch [46] reported that the structure of the enzyme was largely α-helical although there was also a small region (β-sheet). After treatment with NPs, the intensities of two negative peaks in the CD spectra of HRP decreased (at 0.04, 0.14 and 0.16 mM concentration of Ag-NPs), indicating a loss of the α-helix structure fraction of HRP; this result showed that these treatments caused conformational changes in the secondary structures. Above and below the concentration of 0.08 mM of silver nanoparticles an obvious decrease of the ellipticity was observed. The ellipticity of HRP treated by 0.16 mM NPs gradually decreased which resulted in greater decrease of the two negative peaks, indicating α-helix conformation reduction. This corresponds nicely to the loss of the above HRP residual activity illustrated in Fig. 7. However, when HRP was treated with 0.08, 0.06, 0.10 and 0.12 mM concentrations of Ag-NPs, an increase in the ellipticity was observed indicating the increase in the α-helix contents which is directly related to the enhanced activity of HRP. Table 2 presents the α-helix relative content of HRP, measured immediately after treatment with different concentrations of Ag-NPs at 4°C. After the NPs treatments at 0.08, 0.06, 0.10 and 0.12 mM concentrations of NPs at 55°C for 60 min, the residual α-helix relative content of those samples increased but when the HRP was treated with the concentration of 0.04, 0.14 and 0.16 mM, the decrease in the α-helix and increase in the β-sheet contents were observed. This difference in the responsiveness of the α-helix secondary structure to NPs treatment could account for the above finding that at specific low concentration of NPs, resulted in a greater increase of HRP residual activity with a maximum residual activity at a very specific concentration of NPs. These results suggest that the reduction of HRP activity could also be related to the destruction of HRP secondary structure at the above mentioned concentration Ag-NPs. The complete mechanism of the enhancement of α-helical contents treated in the presence of Ag-NPs is still unknown. There are various views for the enhancement in the α-helices content in various proteins after immobilization with different supports. According to the Hungerford et al. [47], other motifs present in protein; such as β-pleated sheets, poly-L-proline II-like helices and turn may have been converted to α-helices and because of this the increase in the α-helices contents was observed. This change could increase the availability of heme group in the active site of HRP followed by increase in the activity. In another study [48], the relationship between the residual activity of the enzyme and the α-helix was evaluated. In the research article, a complete mechanism for the increase in the α-helical content was mentioned. A linear relationship between the activity of enzyme and an increase in the α-helical content was proposed [48]. Recently, Rocha et al. [49] describes the basic mechanistic approach for the enhancement of α-helix contents of amyloid protein after interaction with surfactants. There was a concentration threshold for ionic surfactants, at which they were able to induce helical structure of mentioned protein.

**Table 2:** Determination of α-helix relative content (%).

Conc. of Ag-NPs	α-helix relative content (%)
0.0 mM control HRP	100
0.04 mM	76
0.06 mM	120
0.08 mM	139
0.10 mM	112
0.12 mM	103
0.14 mM	74
0.16 mM	67

The α-helix relative content (%) was determined as mentioned in the text. The increase in the α-helix relative contents also supports our hypothesis.

Finally, we have proposed a hypothetical approach for the relationship of the structural change and the efficiency activity profile of immobilized HRP enzyme with Ag-NPs. As can be seen in Fig. 6, at 0.06 mM Ag-NPs concentration, HRP starts to unfold to a greater extent in the solution but below this concentration (at 0.04 mM) the native enzyme conformation exist. This finding indicates the existence of a high energy barrier for unfolding in a crowded surface environment. It is also evident from the Fig. 6 that there was a rapid increase in the unfolded fraction at short time to the 0.08 mM concentration of Ag-NPs. After reaching the highest unfolded state (at which the maximum activity was observed), a much slower renaturation of HRP was observed (Table 1).The slower renaturation of the HRP enzyme is possibly due to the reorientation of adsorbed molecules at the Ag-NPs surface. The equilibration time was found to be dependable on concentrations of Ag-NPs. Therefore **we** can be attribute three phases for the protein unfolding on the surface of Ag-NPs. The initial phase, which took place for concentration up to 0.08 mM of Ag-NPs, is believed to involve rapid unfolding of protein molecules on the surface after its adsorption. Because of the lower energy barrier due to the large surface area available for the protein molecules at lower concentration of Ag-NPs, the process took place at a very short time as indicated by the rapid increase in the unfolded fraction. Since the initial timescale was of the order of minutes, experimental measurements of unfolding within that timescale could not be made and therefore, a jump was observed in the unfolding of the protein. This rapid jump was found to be larger for the low Ag-NPs concentration (up to 0.08 mM). As the concentration of the Ag-NPs increases the extent of the initial jump decreased (Table 1). The extent of renaturation was observed at second phase in the interaction study. In the third phase, the unfolding of the protein started to decrease with increasing concentration of Ag-NPs. At higher concentration (that is in a more crowded environment), protein-protein interaction become important at the surface because of the smaller distance between neighboring protein molecules. However, at lower surface concentrations, protein-protein interaction would become significant only after the distance between neighboring molecules. We can explain this as at low surface concentration, the predominant molecular interaction is between protein and the surface of the Ag-NPs, which induce the unfolding of protein molecules without energy barrier due to the freely available space. However, at higher surface concentration of Ag-NPs, the unfolding behavior of the protein molecules is strictly limits by the interaction between protein molecules. In other words, in a crowded environment, the extension of the protein structure is limited by the physical space and the energy barrier which arises from the interaction with other neighboring protein molecules. Apparently, this energy barrier not only decreases the extent of unfolding but also decreases the unfolding rate at higher surface concentration followed by decrease in the activity of the HRP enzyme. Recently, the same mechanistic approach was evaluated by another research group [50]. In the research article a fungal protease was immobilized on gold nanoparticles and the enhancement in the activity was also observed.

### Conclusion

In this study, Ag-NPs were synthesized using a simple chemical reduction method and were characterized by U.V-vis, FT-IR and TEM analysis. The interaction between HRP and NPs was evaluated by FT-IR, UV-vis, fluorescence and CD spectroscopic studies. The analysis of CD spectra indicated that the *α*-helical content increases considerably in the presence of low concentration of Ag-NPs. In comparison with native enzyme, NP-bound enzyme revealed significant improvement in the activity and stability which could henceforth contribute to its better use in various analytical, diagnostic and clinical applications. Furthermore, the reactors containing such Ag-NP adsorbed enzyme could be exploited for the degradation of various aromatic pollutants present in wastewater in batch systems as well as in continuous systems in the near future. Finally, we reported here the simple, economically viable and environmentally friendly method for the synthesis of Ag-NPs.

Enzyme dysfunction is related to many diseases. It is desirable to be able to regulate enzyme conformation and function. NPs could be selected as a carrier for enzymes and control their functions after surface modifications, providing a promising strategy for therapy. Moreover, we could apply these NPs for the detection of cancerous cell in a mix population. Cancer cell surface-specific antibodies could be generated in mice against induced cancer, conjugate with synthesized Ag-NPs and could be exploited to distinguish between normal and cancerous cells. The tagging of antibody conjugated with Ag-NPs onto the tumor cell surface would give a clue as novel nano-carriers not only for the antibodies but for other tag materials also; this approach could be exploited for “target specific drug delivery”. Thus, use of nanoparticles as a carrier could be extended for the rapid, specific and cost effective detection of various cancers, hormones, pathogenic microbes and their toxins if a specific tagging material is available in future.
